# Supplementary material for: STYXL1 regulates CCT complex assembly and flagellar tubulin folding in sperm formation
Source: Nat Commun. 2024 Jan 2;15:44. doi: 10.1038/s41467-023-44337-1 (PMC10761714; doi:10.1038/s41467-023-44337-1)
Supplement: Supplementary file 1 — Supplementary Information [file 41467_2023_44337_MOESM1_ESM.pdf]

STYXL1 regulates CCT complex assembly and flagellar tubulin folding in sperm formation

Yu Chen[1, 3, #], Mengjiao Luo[1, #], Haixia Tu[1, 2, #], Yaling Qi[1, #], Yueshuai Guo[1], Xiangzheng Zhang[1], Yiqiang Cui[1], Mengmeng Gao[1], Xin Zhou[1], Tianyu Zhu[1], Hui Zhu[1], Chenghao Situ[1,*], Yan Li[2,*], Xuejiang Guo[1,*]

[1] Department of Histology and Embryology, State Key Laboratory of Reproductive Medicine and Offspring Health, Nanjing Medical University, Nanjing, 211166, China.

[2] Department of Clinical Laboratory, Sir Run Run Hospital, Nanjing Medical University, Nanjing, 211166, China.

[3] Medical Research Center, Changzhou Maternal and Child Health Care Hospital, Changzhou Medical Center, Nanjing Medical University, Changzhou, 213000, China.

# Equal contribution

* To whom correspondence should be addressed. Tel: +86-25-86869383; Fax: +86-25-86869383; Email: guo_xuejiang@njmu.edu.cn (X.G.), yanli@njmu.edu.cn (Y.L.), situchenghao@njmu.edu.cn (C.S.)

Supplementary information

Supplementary Fig. 1 Sequence conservation and expression analysis of STYXL1.

Supplementary Fig. 2 The phenotype analysis of *Styxl1*$^{\Delta 4bp/\Delta 4bp}$ male mice.

Supplementary Fig. 3 Immunofluorescence analysis of spermiogenesis after deletion of *Styxl1*.

Supplementary Fig. 4 Quantitative proteomic analysis of *Styxl1*$^{-/-}$ sperm.

Supplementary Fig. 5 GST pull down assay of GST-fused mouse STYXL1 protein.

Supplementary Fig. 6 Extracted ion chromatograms from protein quantification measurements by PRM.

Supplementary Table 1. Antibodies used in this study.

Supplementary Table 2. Primers used in qRT-PCR.

Supplementary Table 3. Heavy peptide sequences used in PRM quantification.

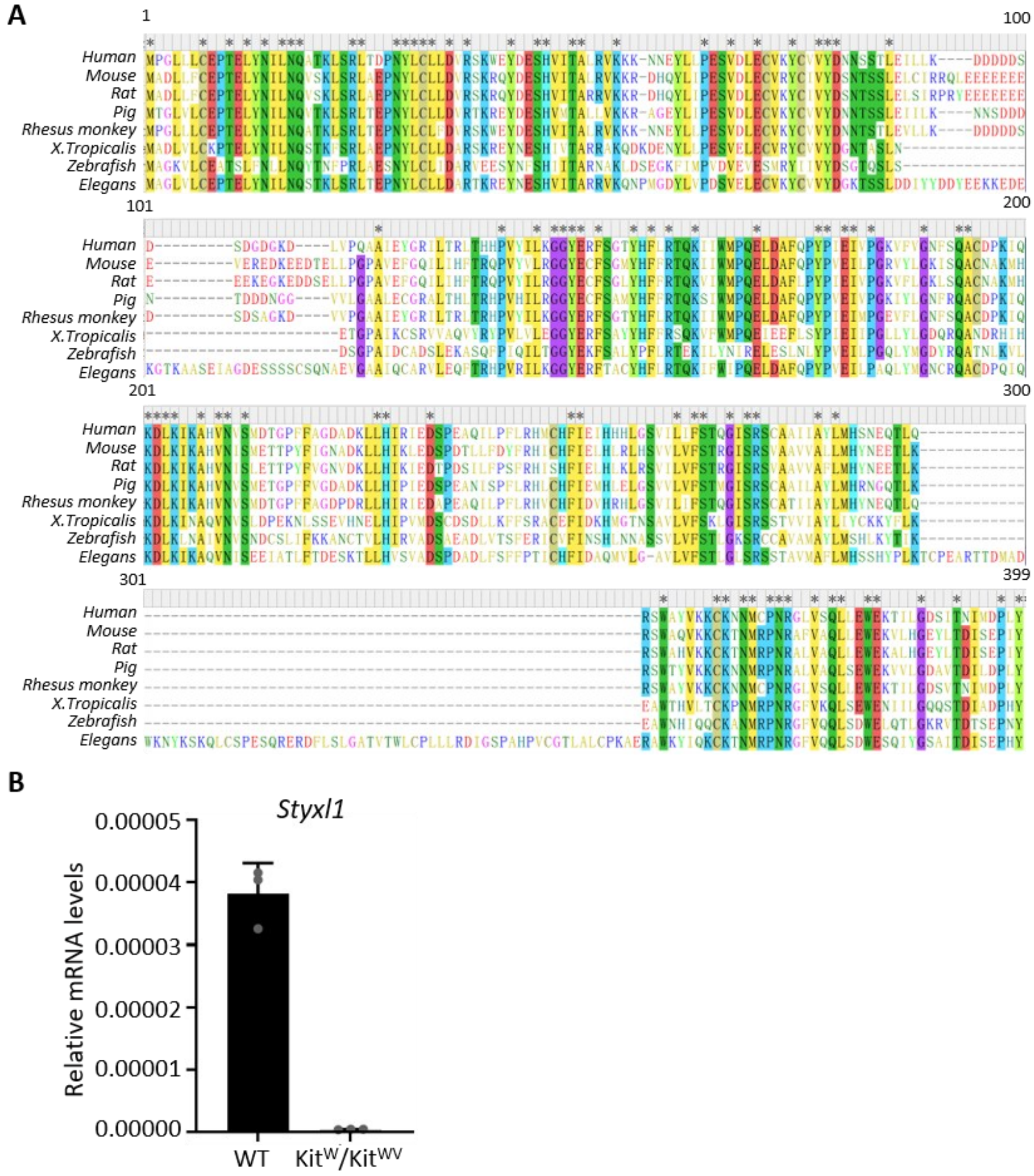

Supplementary Fig.1 Sequence conservation and expression analysis of STYXL1. **(A)** Multiple sequence alignment of STYXL1 proteins from different species. **(B)** *Styxl1* mRNA expression levels quantified in adult WT and Kit^{W}/Kit^{WV} testes by qRT-PCR (n=3 mice per group). Data are presented with the mean ± SD. Source data are provided as a Source Data file.

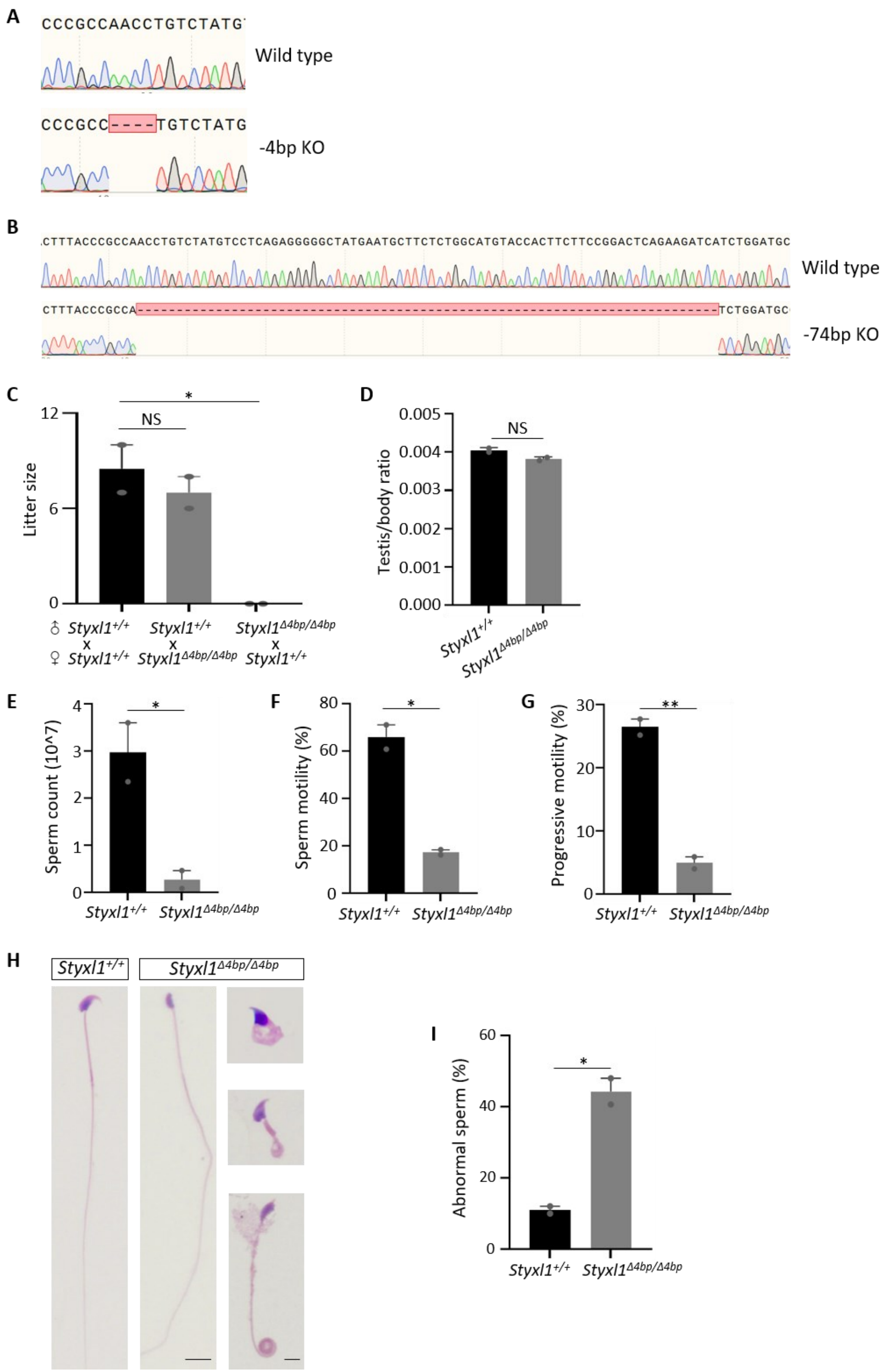

Supplementary Fig. 2 The phenotype analysis of *Styxl1*$^{\Delta 4bp/\Delta 4bp}$ male mice. (A-B) Sanger sequencing showing 4 bp and 74 bp deletion in exon 5 of the *Styxl1* gene. **(C)**

Litter size of adult *Styxl1*$^{\Delta4bp/\Delta4bp}$ male and *Styxl1*$^{\Delta4bp/\Delta4bp}$ female mice (*n*=2 mice per group). NS *p*=0.5583 and **p*=0.0170 using one-way ANOVA followed by Dunnett's multiple comparisons test. **(D)** Statistics analysis of adult *Styxl1*$^{+/+}$ and *Styxl1*$^{\Delta4bp/\Delta4bp}$ testis/body weight ratio (*n*=2 mice per group). NS *p*=0.0695 using two-tailed Student's t-test. **(E-G)** Quantitative analysis of sperm count (**p*=0.0107 using two-tailed Student's t-test) **(E),** sperm motility (**p*=0.0115 using two-tailed Student's t-test) **(F)** and progressive motility (***p*=0.0053 using two-tailed Student's t-test) **(G)** of adult *Styxl1*$^{+/+}$ and *Styxl1*$^{\Delta4bp/\Delta4bp}$ mice (*n*=2 mice per group)**. (H-I)** The morphologies of adult *Styxl1*$^{\Delta4bp/\Delta4bp}$ sperm by H&E staining (*n*=3 biologically independent samples) **(H)** and the percentage of sperm abnormalities (*n*=2 mice per group**,** **p*=0.0129 using two-tailed Student's t-test) **(I).** Scale bar:5μm. NS, not significant; *, $p < 0.05$; **, $p < 0.01$; ***, $p < 0.001$. Data are presented as the mean ± SEM. Source data are provided as a Source Data file.

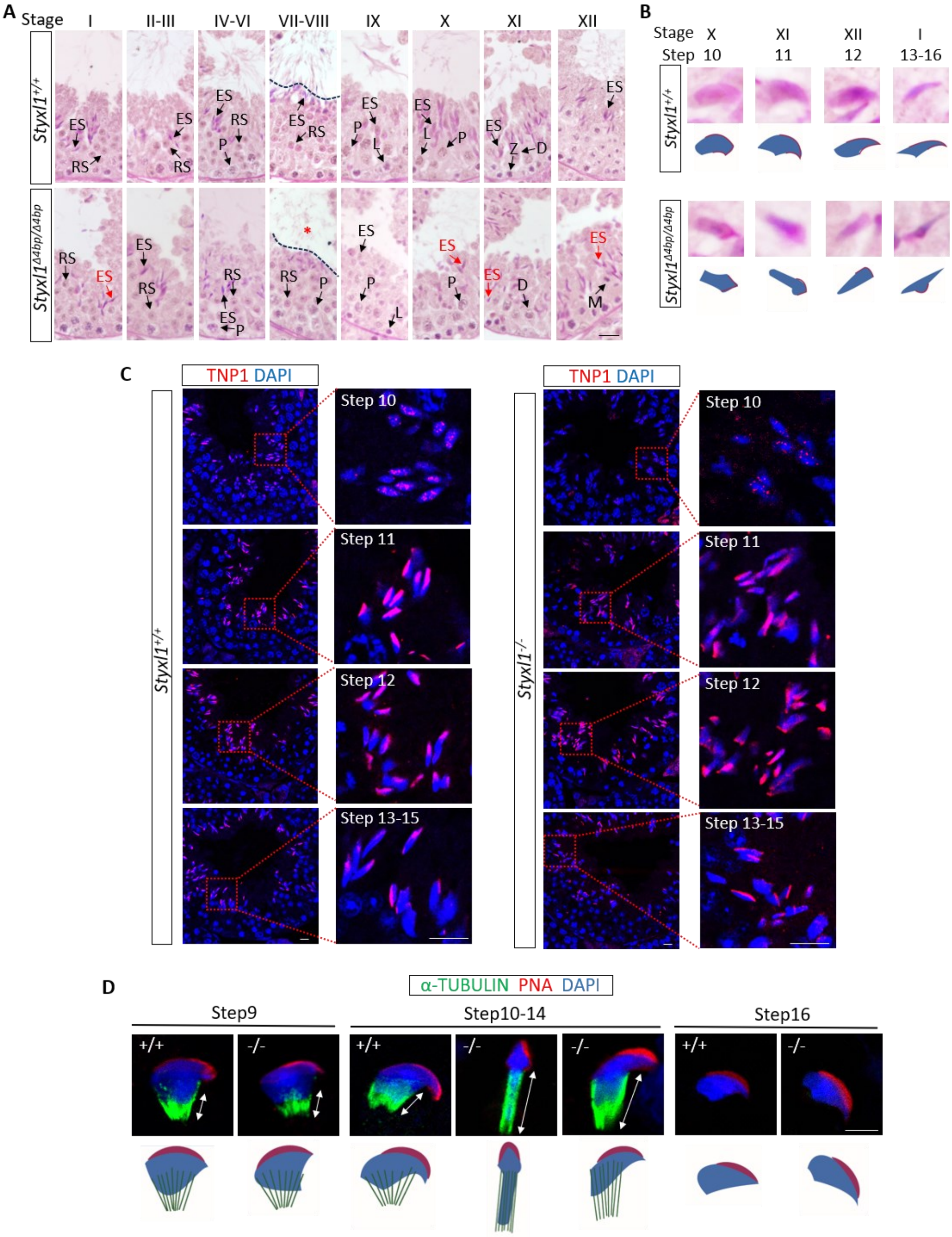

Supplementary Fig. 3 Immunofluorescence analysis of spermiogenesis after deletion of *Styxl1*. **(A)** Different stages of seminiferous tubules in PAS-stained adult *Styxl1*$^{+/+}$ and *Styxl1*$^{\Delta4bp/\Delta4bp}$ testes. The red arrow indicates abnormal nuclei of elongating spermatids. Asterisk indicates defects of sperm tails. L, leptotene; Z, zygotene; P, pachytene; D, diplotene; RS, round spermatid; ES, elongated spermatid. M, metaphase. Scale bar: 25μm. **(B)** Enlarged pictures of different steps of adult

Styxl1$^{+/+}$ and *Styxl1*$^{\Delta 4bp/\Delta 4bp}$ elongated spermatids pointed by arrows in **(A)** were presented. Schematic diagrams were denoted at bottom. **(C)** Immunofluorescence staining of TNP1 (red) in adult *Styxl1*$^{+/+}$ and *Styxl1*$^{-/-}$ seminiferous tubules with nuclei stained by DAPI (blue). Scale bar: 10μm. **(D)** Immunofluorescence of α-TUBULIN (green) in different steps of spermatids containing manchette from adult *Styxl1*$^{+/+}$ and *Styxl1*$^{-/-}$ testes with nuclei stained by DAPI (blue). Scale bar: 5μm. *n*=3 biologically independent samples were included in each group (A, C, D).

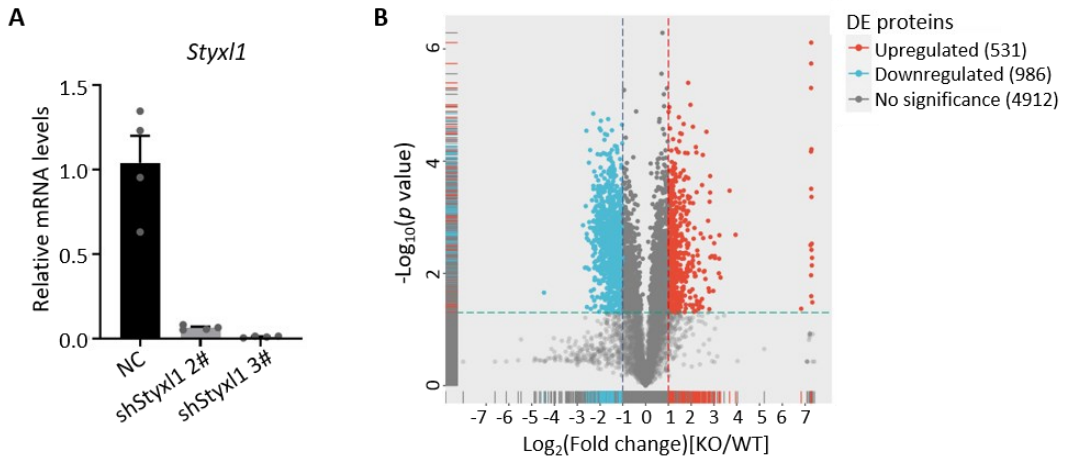

Supplementary Fig. 4 Quantitative proteomic analysis of *Styxl1$^{-/-}$* sperm. (A) The relative mRNA levels of *Styxl1* in control and *Styxl1* shRNA treated groups (n=4 biological replicates per group). Data are presented as the mean ± SEM. **(B)** The volcano plot of quantified proteins between adult *Styxl1$^{+/+}$* and *Styxl1$^{-/-}$* sperm. The cutoff values (p<0.05 and Foldchange>2) were utilized to identify significantly differentially expressed proteins. Source data are provided as a Source Data file.

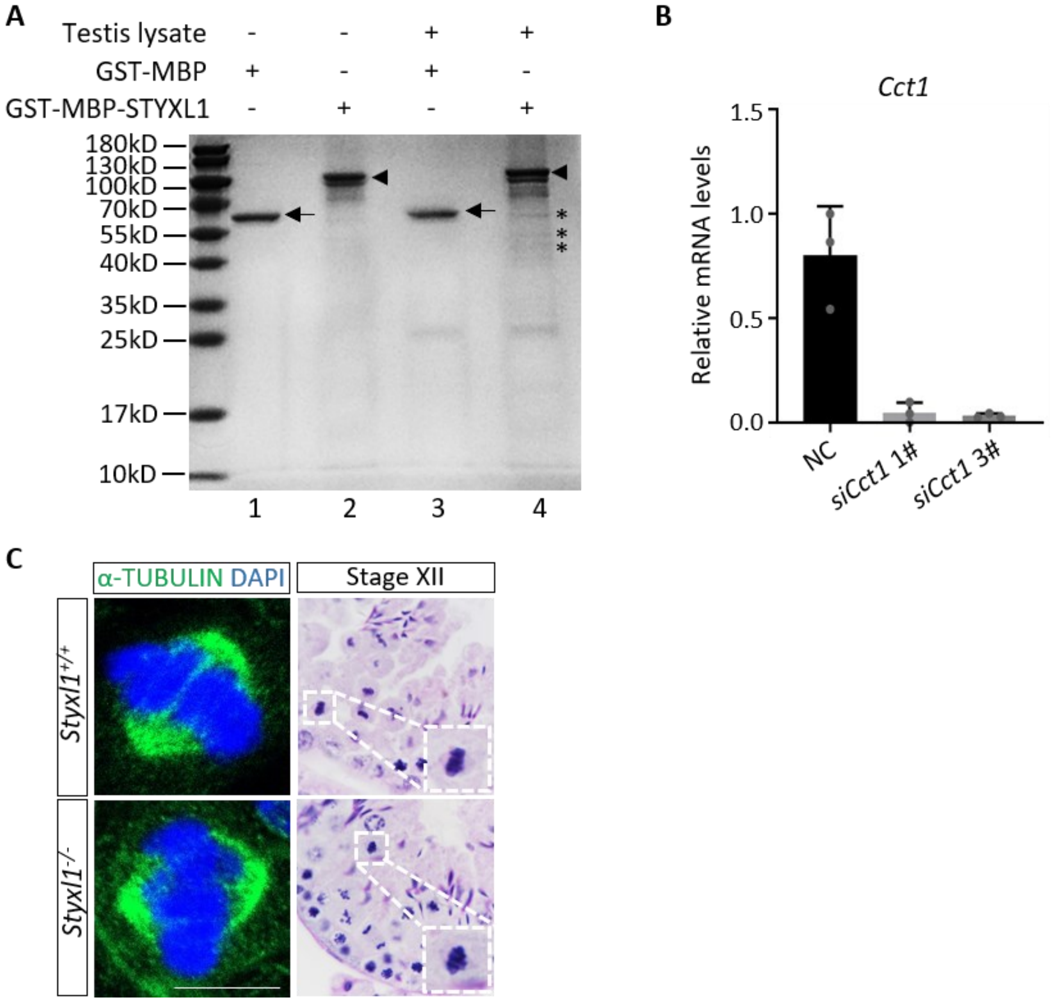

Supplementary Fig. 5 GST pull down assay of GST-fused mouse STYXL1 protein. **(A)** SDS-PAGE analysis and Coomassie blue staining of the eluates from GST-beads. *n*=3 biologically independent samples were included. The arrow and arrowhead indicate the bands of GST-MBP and GST-MBP-STYXL1 protein, respectively. The asterisks indicate the bands that were differentially pulled down by GST–MBP-STYXL1 beads from testis lysate compared with GST-MBP with lysate and GST-MBP-STYXL1 without lysate. **(B)** The relative mRNA levels of *Cct1* in control and *Cct1* siRNA treated groups (*n*=3 biological replicates per group). Data are presented as the mean ± SD. **(C)** Immunofluorescent staining of α-tubulin (green) and H&E-staining of metaphase spermatocytes in adult *Styxl1*$^{+/+}$ and *Styxl1*$^{-/-}$ testes. *n*=3 biologically independent samples were included. Nuclei were stained with DAPI (blue). Insets indicate zoom-in views of the boxed metaphase spermatocytes. Scale bar: 10μm. Source data are provided as a Source Data file.

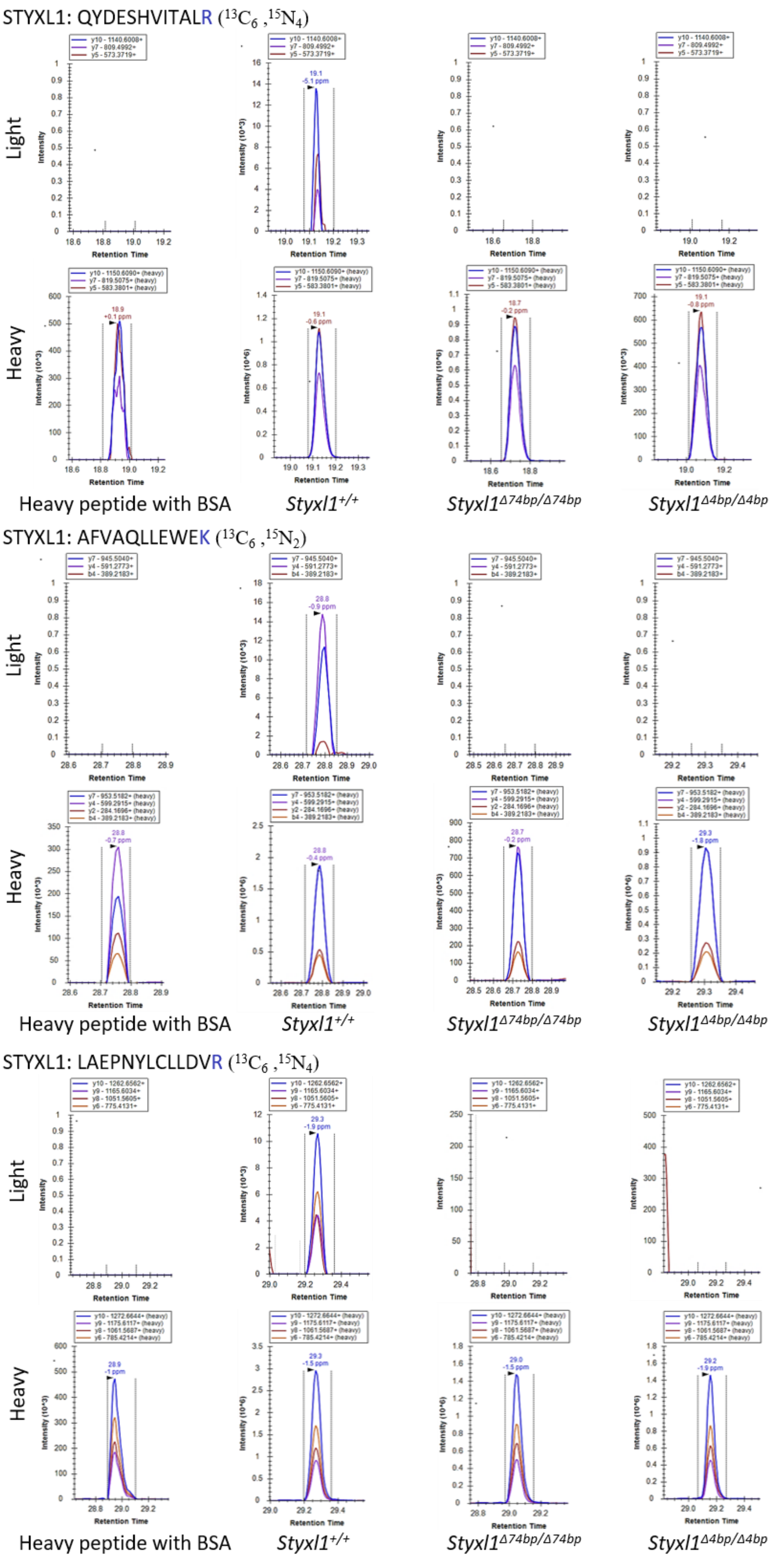
STYXL1: QYDESHVITALR ($^{13}C_6$, $^{15}N_4$)
Light
Heavy
Heavy peptide with BSA
$Styxl1^{+/+}$
$Styxl1^{\Delta 74bp/\Delta 74bp}$
$Styxl1^{\Delta 4bp/\Delta 4bp}$
STYXL1: AFVAQLLEWEK ($^{13}C_6$, $^{15}N_2$)
Light
Heavy
Heavy peptide with BSA
$Styxl1^{+/+}$
$Styxl1^{\Delta 74bp/\Delta 74bp}$
$Styxl1^{\Delta 4bp/\Delta 4bp}$
STYXL1: LAEPNYLCLLDVR ($^{13}C_6$, $^{15}N_4$)
Light
Heavy
Heavy peptide with BSA
$Styxl1^{+/+}$
$Styxl1^{\Delta 74bp/\Delta 74bp}$
$Styxl1^{\Delta 4bp/\Delta 4bp}$
Retention Time
Intensity

Supplementary Fig. 6 Extracted ion chromatograms from protein quantification measurements by PRM. Representative extracted ion chromatograms of three peptides from STYXL1 protein in quantificative measurements by PRM. Heavy peptides with BSA were used as a negative control.

Supplementary Table 1. Antibodies used in this study.

Antibodies	Brand	Cat No.
DDDDK-tag	MBL	PM020
α-TUBULIN	Abways	P68366
TNP1	Proteintech	17178-1-AP
AC-TUBULIN	Sigma	T6793
HA-tag	Sigma	H6908
SLC2A3	Proteintech	20403-1-AP
AKAP4	Proteintech	24986-1-AP
CCT1	Proteintech	10320-1-AP
CCT2	Proteintech	24896-1-AP
CCT3	Proteintech	10571-1-AP
CCT4	Proteintech	21524-1-AP
CCT5	Proteintech	11603-1-AP
CCT6	Proteintech	19793-1-AP
CCT7	Proteintech	15994-1-AP
CCT8	Proteintech	12263-1-AP
PRM2	Biarpatchbio	Mab-Hup2B-150
β-TUBULIN	Abclonal	AC021
GAPDH	Abways	AB0036
PNA	VECTORLABS	RL-1072

Supplementary Table 2. Primers used in qRT-PCR.

Gene	Forward primer (5'-3')	Reverse primer (5'-3')
Styxl1	CTGCTTTTCTGCGAGCCAAC	TGTCGTTTTGATCGGACATCC
Cct1	CCGCTCCCAGAATGTTATGG	CGGGATGTTCTACCTCCAGT

Supplementary Table 3. Heavy peptide sequences used in PRM quantification.

Gene Names	Heavy Peptide Sequence
STYXL1	QYDESHVITAL**R** ($^{13}C_6$,$^{15}N_4$)
STYXL1	AFVAQLLEWE**K** ($^{13}C_6$,$^{15}N_2$)
STYXL1	LAEPNYLCLLDV**R** ($^{13}C_6$,$^{15}N_4$)
