# Supplementary material for: STYXL1 regulates CCT complex assembly and flagellar tubulin folding in sperm formation
Source: Nat Commun. 2024 Jan 2;15:44. doi: 10.1038/s41467-023-44337-1 (PMC10761714; doi:10.1038/s41467-023-44337-1)
Supplement: Supplementary file 3 — Description of Additional Supplementary Files [file 41467_2023_44337_MOESM3_ESM.pdf]

Description of Additional Supplementary Files

File Name: Supplementary Data 1
Description: Proteomics profiling of Styxl1+/+ and Styxl1-/- sperm.

File Name: Supplementary Movie 1
Description: Sperm motility analysis video of Styxl1+/+ and Styxl1-/- sperm.
