# Supplementary material for: STYXL1 regulates CCT complex assembly and flagellar tubulin folding in sperm formation
Source: Nat Commun. 2024 Jan 2;15:44. doi: 10.1038/s41467-023-44337-1 (PMC10761714; doi:10.1038/s41467-023-44337-1)
Supplement: Supplementary file 6 — Reporting Summary [file 41467_2023_44337_MOESM6_ESM.pdf]

nature portfolio

Corresponding author(s): Xuejiang Guo, Yan Li, Chenghao Situ

Last updated by author(s): Nov 1, 2023

Reporting Summary

Nature Portfolio wishes to improve the reproducibility of the work that we publish. This form provides structure for consistency and transparency in reporting. For further information on Nature Portfolio policies, see our Editorial Policies and the Editorial Policy Checklist.

Statistics

For all statistical analyses, confirm that the following items are present in the figure legend, table legend, main text, or Methods section.

n/a	Confirmed	
☐	☒	The exact sample size (*n*) for each experimental group/condition, given as a discrete number and unit of measurement
☐	☒	A statement on whether measurements were taken from distinct samples or whether the same sample was measured repeatedly
☐	☒	The statistical test(s) used AND whether they are one- or two-sided<br>*Only common tests should be described solely by name; describe more complex techniques in the Methods section.*
☒	☐	A description of all covariates tested
☒	☐	A description of any assumptions or corrections, such as tests of normality and adjustment for multiple comparisons
☐	☒	A full description of the statistical parameters including central tendency (e.g. means) or other basic estimates (e.g. regression coefficient) AND variation (e.g. standard deviation) or associated estimates of uncertainty (e.g. confidence intervals)
☐	☒	For null hypothesis testing, the test statistic (e.g. *F*, *t*, *r*) with confidence intervals, effect sizes, degrees of freedom and *P* value noted<br>*Give P values as exact values whenever suitable.*
☒	☐	For Bayesian analysis, information on the choice of priors and Markov chain Monte Carlo settings
☒	☐	For hierarchical and complex designs, identification of the appropriate level for tests and full reporting of outcomes
☒	☐	Estimates of effect sizes (e.g. Cohen's *d*, Pearson's *r*), indicating how they were calculated

Our web collection on statistics for biologists contains articles on many of the points above.

Software and code

Policy information about availability of computer code

Data collection	Mass spectrometry data were acquired by Xcalibur 4.3.73.11 (Thermo Fisher Scientific) on Orbitrap Fusion Lumos (Thermo Fisher Scientific, USA).
Data analysis	Skyline Daily software (http://proteome.gs.washington.edu/software/skyline) was used to process the PRM data. For TMT experiment, all raw files were searched with MaxQuant software (Version 2.2.0.0) using mouse protein sequences obtained from the Universal Protein Resource database (UniProt, release 2022_01). Mean and SD/SEM were calculated using GraphPad Prism 9. The schematic diagrams were drawn by Adobe Illustrator 2020.

For manuscripts utilizing custom algorithms or software that are central to the research but not yet described in published literature, software must be made available to editors and reviewers. We strongly encourage code deposition in a community repository (e.g. GitHub). See the Nature Portfolio guidelines for submitting code & software for further information.

Data

Policy information about availability of data

All manuscripts must include a data availability statement. This statement should provide the following information, where applicable:

- Accession codes, unique identifiers, or web links for publicly available datasets
- A description of any restrictions on data availability
- For clinical datasets or third party data, please ensure that the statement adheres to our policy

The MS proteomics data generated in this study have been deposited in the PRIDE database under accession code PXD046203.

Research involving human participants, their data, or biological material

Policy information about studies with human participants or human data. See also policy information about sex, gender (identity/presentation), and sexual orientation and race, ethnicity and racism.

Reporting on sex and gender	not applicable
Reporting on race, ethnicity, or other socially relevant groupings	not applicable
Population characteristics	not applicable
Recruitment	not applicable
Ethics oversight	not applicable

Note that full information on the approval of the study protocol must also be provided in the manuscript.

Field-specific reporting

Please select the one below that is the best fit for your research. If you are not sure, read the appropriate sections before making your selection.

☒ Life sciences ☐ Behavioural & social sciences ☐ Ecological, evolutionary & environmental sciences

For a reference copy of the document with all sections, see nature.com/documents/nr-reporting-summary-flat.pdf

Life sciences study design

All studies must disclose on these points even when the disclosure is negative.

Sample size	Sample sizes were not predetermined. Experiments were conducted in multiple replicates as stated in the figure legends. Most experiments were performed at least three times independently. For 4bp mutant mouse line, a few data were based on two replicates, showing similar results to those of 174bp mutant mouse line. The sample size was determined based on previous experience and reports for each experiment to yield high power to detect specific effects.
Data exclusions	No data were excluded from the analyses.
Replication	All experiments were conducted multiple times (as stated in the figure legends). The results were reproducible.
Randomization	Animals were chosen according to correct genotypes.
Blinding	Styxl1 knockout mice were used as experimental groups and wildtype mice are used as control groups. Therefore, blinding was not relevant to our study.

Reporting for specific materials, systems and methods

We require information from authors about some types of materials, experimental systems and methods used in many studies. Here, indicate whether each material, system or method listed is relevant to your study. If you are not sure if a list item applies to your research, read the appropriate section before selecting a response.

Materials & experimental systems

n/a	Involved in the study
☐	☒ Antibodies
☐	☒ Eukaryotic cell lines
☒	☐ Palaeontology and archaeology
☐	☒ Animals and other organisms
☒	☐ Clinical data
☒	☐ Dual use research of concern
☒	☐ Plants

Methods

n/a	Involved in the study
☒	☐ ChIP-seq
☒	☐ Flow cytometry
☒	☐ MRI-based neuroimaging

Antibodies

Antibodies used

DDDDK-tag, MBL, PM020; α-TUBULIN, Abways, P68366; TNP1, Proteintech, 17178-1-AP; AC-TUBULIN, Sigma, T6793; HA-tag, Sigma, H6908; SLC2A3, Proteintech, 20403-1-AP; AKAP4, Proteintech, 24986-1-AP; CCT1, Proteintech, 10320-1-AP; CCT2, Proteintech, 24896-1-AP; CCT3, Proteintech, 10571-1-AP; CCT4, Proteintech, 21524-1-AP; CCT5, Proteintech, 11603-1-AP; CCT6, Proteintech, 19793-1-AP; CCT7, Proteintech, 15994-1-AP; CCT8, Proteintech, 12263-1-AP; PRM2, Biarpatchbio, Mab-Hup2B-150; β-TUBULIN, Abclonal, AC021; GAPDH, Abways, AB0036; PNA, VECTORLABS, RL-1072

Validation

Each antibody was validated according to their manufacturer's website and provided in the manuscript.

Eukaryotic cell lines

Policy information about cell lines and Sex and Gender in Research

Cell line source(s)

The HEK293T (ATCC, CRL-3216) and NIH3T3 cells (ATCC, CRL-1658) were used in this study.

Authentication

Both the cell lines used were authenticated using STR profiling test by Shanghai Biowing Applied Biotechnology Co., Ltd.

Mycoplasma contamination

The cell lines were tested negative for mycoplasma contamination using TransDetect PCR Mycoplasma detection kit (Transgen, Beijing, China).

Commonly misidentified lines
(See ICLAC register)

No commonly misidentified cell lines were used.

Animals and other research organisms

Policy information about studies involving animals; ARRIVE guidelines recommended for reporting animal research, and Sex and Gender in Research

Laboratory animals

C57BL/6 mice, 8 weeks of age, male. C57BL/6J mice were housed in a specific-pathogen-free animal facility, provided with standard food, given free access to hypochlorous weak-acid water, and maintained under a 12h–12h light–dark cycle with the temperature 24 ±1°C and humidity at 50±10%.

Wild animals

The study did not involve wild animals.

Reporting on sex

Fertility test showed that Styxl1–/– male mice were completely infertile , thus male mice are considered in study design.

Field-collected samples

The study did not involve samples collected from the field.

Ethics oversight

All animal experiments were approved by the Animal Welfare Committee of Nanjing Medical University.

Note that full information on the approval of the study protocol must also be provided in the manuscript.

Plants

Seed stocks	not applicable
Novel plant genotypes	not applicable
Authentication	not applicable
